# Cognitive Neuroscience of Attention Deficit Hyperactivity Disorder (ADHD) and Its Clinical Translation

**DOI:** 10.3389/fnhum.2018.00100

**Published:** 2018-03-29

**Authors:** Katya Rubia

**Affiliations:** Child & Adolescent Psychiatry, Institute of Psychiatry, Psychology & Neuroscience (IoPPN), King’s College London, London, United Kingdom

**Keywords:** Attention Deficit Hyperactivity Disorder (ADHD), functional magnetic resonance imaging (fMRI), pattern recognition analysis, executive functions, fMRI-Neurofeedback, transcranial direct current stimulation, transcranial magnetic stimulation, neuromodulation

## Abstract

This review focuses on the cognitive neuroscience of Attention Deficit Hyperactivity Disorder (ADHD) based on functional magnetic resonance imaging (fMRI) studies and on recent clinically relevant applications such as fMRI-based diagnostic classification or neuromodulation therapies targeting fMRI deficits with neurofeedback (NF) or brain stimulation. Meta-analyses of fMRI studies of executive functions (EFs) show that ADHD patients have cognitive-domain dissociated complex multisystem impairments in several right and left hemispheric dorsal, ventral and medial fronto-cingulo-striato-thalamic and fronto-parieto-cerebellar networks that mediate cognitive control, attention, timing and working memory (WM). There is furthermore emerging evidence for abnormalities in orbital and ventromedial prefrontal and limbic areas that mediate motivation and emotion control. In addition, poor deactivation of the default mode network (DMN) suggests an abnormal interrelationship between hypo-engaged task-positive and poorly “switched off” hyper-engaged task-negative networks, both of which are related to impaired cognition. Translational cognitive neuroscience in ADHD is still in its infancy. Pattern recognition analyses have attempted to provide diagnostic classification of ADHD using fMRI data with respectable classification accuracies of over 80%. Necessary replication studies, however, are still outstanding. Brain stimulation has been tested in heterogeneously designed, small numbered proof of concept studies targeting key frontal functional impairments in ADHD. Transcranial direct current stimulation (tDCS) appears to be promising to improve ADHD symptoms and cognitive functions based on some studies, but larger clinical trials of repeated stimulation with and without cognitive training are needed to test clinical efficacy and potential costs on non-targeted functions. Only three studies have piloted NF of fMRI-based frontal dysfunctions in ADHD using fMRI or near-infrared spectroscopy, with the two larger ones finding some improvements in cognition and symptoms, which, however, were not superior to the active control conditions, suggesting potential placebo effects. Neurotherapeutics seems attractive for ADHD due to their safety and potential longer-term neuroplastic effects, which drugs cannot offer. However, they need to be thoroughly tested for short- and longer-term clinical and cognitive efficacy and their potential for individualized treatment.

## Introduction

Attention Deficit Hyperactivity Disorder (ADHD) is characterized by symptoms of age-inappropriate inattention, hyperactivity and impulsivity (American Psychiatric Association, 2000). ADHD is one of the most prevalent childhood disorders with a worldwide prevalence of around 7% with problems persisting into adulthood in a substantial proportion of children and is associated with poor academic and social outcomes (Thomas et al., 2015).

Meta-analyses of structural volumetric studies in ADHD have shown deficits most prominently in subcortical regions such as the basal ganglia and insula (Nakao et al., 2011; Norman et al., 2016). The largest recent meta- and mega-analysis of subcortical structural imaging studies across 23 sites including more than 1713 ADHD patients and over 1500 controls, found additional volume reductions besides the basal ganglia in limbic areas such as amygdala and hippocampus (Hoogman et al., 2017). Abnormalities in ventromedial frontal regions, however, have also been observed in large-numbered meta-analyses (Norman et al., 2016; Rubia et al., 2016) and there is evidence for a delay in cortical thickness maturation in frontal, temporal and parietal regions (Shaw et al., 2007, 2012). In addition to the gray matter structural deficits, white matter tracts have also been found to be impaired in the disorder, most prominently fronto-striato-cerebellar as well as fronto-posterior and interhemispheric tracts (Chen et al., 2016).

Several reviews have been published on the neuroimaging findings in ADHD (Rubia, 2011; Rubia et al., 2014a; Faraone et al., 2015). This review is focusing particularly on the cognitive neuroscience of the disorder, by reviewing the most consistent findings of functional magnetic resonance imaging (fMRI) studies in ADHD during cognitive and emotional tasks. It also reviews the emerging field of translational cognitive neuroscience in ADHD which has pioneered potential clinical applications of neuroimaging, such as using neuroimaging data for diagnostic classification of the disorder or as targets for treatment. The review will hence discuss recent attempts to use fMRI data to provide more objective diagnostic classifications for ADHD or the use of fMRI biomarkers as targets for imaging based neuromodulation treatments such as self-regulation training with Neurofeedback (NF) using fMRI or near infrared spectroscopy (NIRS) or brain stimulation using magnetic or direct current brain stimulation. Both neuromodulation therapies, NF and brain stimulation, aim to improve ADHD symptoms and cognition by targeting the underlying regional dysfunctions that are thought to be underlying the condition. Translational cognitive neuroscience in ADHD is still very much in its childhood, but has provided promising results so far.

The literature search for this review used scientific databases such as www.pubmed.com, and ISI web of science^1^ and was conducted up to 20th of January 2018. Search terms included “ADHD”, “Attention Deficit Hyperactivity Disorder”, or “ADD” or “Attention Deficit Disorder” combined with one of the following terms: “fMRI”, “MRI”, “(multivariate)pattern recognition analysis”, “support vector machine”, “machine learning”, “brain stimulation”, “transcranial magnetic stimulation” or “TMS”, “transcranial direct current stimulation” or “tDCS” and “NIRS-NF”. Additional references were searched in the resulting publications, including reviews and meta-analyses.

## Cognitive Neuroscience of ADHD

ADHD patients have deficits in higher-level cognitive functions necessary for mature adult goal-directed behaviors, in so-called “executive functions” (EFs), that are mediated by late developing fronto-striato-parietal and fronto-cerebellar networks (Rubia, 2013). The most consistent deficits are in so-called “cool” EF such as motor response inhibition, working memory (WM), sustained attention, response variability and cognitive switching (Willcutt et al., 2008; Rubia, 2011; Pievsky and McGrath, 2018) as well as in temporal processing (i.e., motor timing, time estimation and temporal foresight), with most consistent deficits in time discrimination and estimation tasks (Rubia et al., 2009a; Noreika et al., 2013). However, impairment has also been found in so-called “hot” EF functions of motivation control and reward-related decision making, as measured in temporal discounting and gambling tasks, with, however, more inconsistent findings (Willcutt et al., 2008; Noreika et al., 2013; Plichta and Scheres, 2014). Evidence for cognitive deficits is more consistent in children than adolescents or adults with ADHD (Groen et al., 2013; Pievsky and McGrath, 2018). Last, there is considerable heterogeneity in cognitive impairments, with some patients not showing impairments or only in some cognitive domains, which may be underpinned by different pathophysiological pathways (Sonuga-Barke, 2003; Nigg et al., 2005; Sonuga-Barke et al., 2010).

## fMRI Studies of Cognitive Functions in ADHD

Since the advent of fMRI, several hundreds of fMRI studies have been published in ADHD children and adults over the last two decades, the majority of them targeting cognitive functions. The first fMRI studies conducted in very small numbers of ADHD patients found reduced inferior fronto-striatal activation in ADHD children relative to age-matched healthy controls during motor inhibition (Vaidya et al., 1998; Rubia et al., 1999), which has been widely replicated until today and may even be a disorder-specific feature of ADHD relative to other childhood disorders (Rubia et al., 2014a; Sebastian et al., 2014; Norman et al., 2016). However, more widespread dysfunctions have been observed in ADHD, involving not only the lateral prefrontal cortex and its connections to the basal ganglia, but also medial frontal, cingulate and orbital frontal regions, and the dissociated fronto-parietal, fronto-limbic and fronto-cerebellar networks they form part of Arnsten and Rubia (2012) and Rubia et al. (2014a).

**Figure 1:**
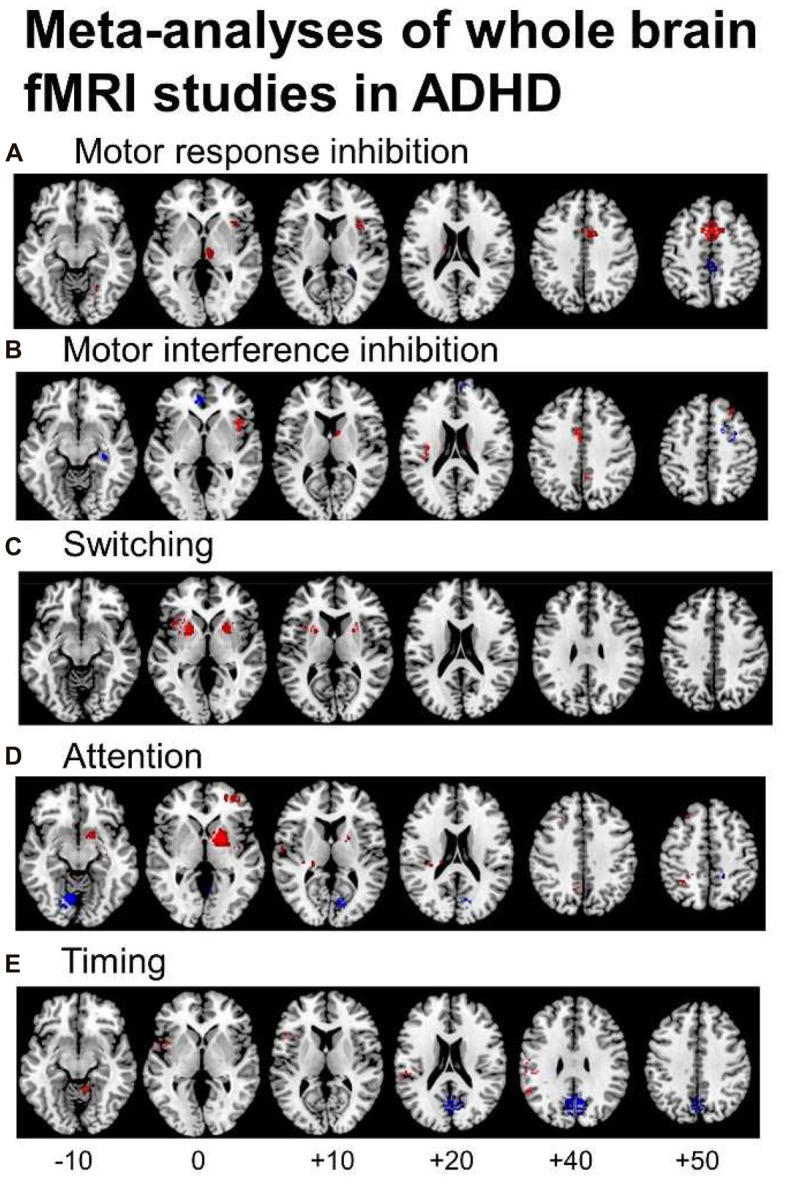
Meta-analyses of functional magnetic resonance imaging (fMRI) studies of Attention Deficit Hyperactivity Disorder (ADHD) patients for different cognitive domains. The meta-analyses show underactivation in ADHD patients in several dissociated fronto-striato-parietal and fronto-cerebellar networks during the respective cognitive domains. **(A)** Representation of the meta-analysis of motor response inhibition tasks alone, where 187 ADHD patients relative to 206 healthy controls showed underactivation in right inferior frontal cortex (IFC), supplementary motor area (SMA), basal ganglia and thalamus. They showed enhanced activation in posterior cingulate (Hart et al., 2013). **(B)** Axial slices of the meta-analysis of interference inhibition tasks alone, where 100 ADHD patients relative to 114 healthy controls had underactivation in right IFC, anterior cingulate cortex (ACC), the basal ganglia and thalamus and enhanced activation relative to healthy controls in rostromedial prefrontal cortex (Hart et al., 2013). **(C)** Axial slices of a meta-analysis of switching tasks (Rubia, 2018) based on three fMRI studies, showing decreased activation in 38 ADHD patients relative to 48 healthy controls in left IFC, and bilateral insula, putamen and globus pallidus. **(D)** Axial slices of the meta-analysis of attention tasks, where 171 ADHD patients showed reduced activation relative to 178 healthy controls in the right dorsal attention network, comprising right dorsolateral prefrontal cortex (DLPFC), the posterior part of the basal ganglia and thalamus, inferior parietal lobe and precuneus (PPC). ADHD patients had enhanced activation relative to controls in cerebellum and occipital regions (Hart et al., 2013). **(E)** Axial slices of the meta-analysis of timing tasks, where 150 ADHD children had reduced activation relative to 145 healthy controls in a predominantly left hemispheric timing network, comprising left IFC, left inferior parietal lobe and right cerebellum. ADHD patients had enhanced activation in a default mode region, the posterior cingulate (Hart et al., 2012). The enhanced activation in anterior and posterior cingulate during motor and interference inhibition and timing tasks likely reflects decreased deactivation of the default mode network (DMN) in ADHD vs. healthy controls.

Several fMRI meta-analyses have been published recently, the majority including fMRI studies using cool EF tasks. They show cognitive domain-dissociated brain dysfunctions in several fronto-striatal, fronto-parietal and fronto-cerebellar networks in ADHD. A meta-analysis of 21 whole-brain fMRI studies of cognitive and motor inhibition, including seven adult and 14 pediatric studies, showed that 287 ADHD patients relative to 320 healthy controls had consistently reduced activation in key regions of motor response inhibition, in right inferior prefrontal cortex (IFC)/anterior insula, the supplementary motor area (SMA), anterior cingulate cortex (ACC), left striatum and right thalamus (Hart et al., 2013; Figure 1A). When inhibition tasks were split into motor response and interference inhibition, the reduced activations were more prominently right-hemispheric and in the SMA for motor response inhibition (Figure 1A), while for tasks of interference inhibition (Figure 1B), left ACC dysfunction was more prominent (Hart et al., 2013), in line with the prominent role of the SMA for motor inhibition (Rae et al., 2014) and the ACC for interference inhibition (Nee et al., 2007), respectively. For switching tasks, where only 3 whole-brain fMRI studies were available, including 38 ADHD patients and 48 healthy controls, reduced activation was observed in left IFC, and in bilateral anterior insula, putamen and globus pallidus (Rubia, 2018; see Figure 1C). The findings of cognitive control related brain dysfunctions were replicated in a more recent meta-analysis including 541 ADHD and 620 healthy control adolescents across 40 fMRI studies of motor and response inhibition and switching which found reduced activation in bilateral IFC/anterior insula, striatum, SMA and superior temporal lobe. The dysfunctions furthermore overlapped with reduced volumes in right anterior insula and putamen (Norman et al., 2016).

Another smaller meta-analysis further separated fMRI studies using Stop and Go/no-go tasks (McCarthy et al., 2014). The Stop task fMRI meta-analysis, based on five pediatric and one adult fMRI studies, confirmed the previous meta-analytical findings that 74 ADHD relative to 102 controls had reduced activation in bilateral IFC/insula, but showed additionally reduced activation in right superior and middle frontal cortices (McCarthy et al., 2014). For the Go/no-go task, 149 ADHD patients had reduced activation relative to 159 healthy controls in predominantly left medial frontal cortex (MFC)/ACC and right caudate cortices (McCarthy et al., 2014), suggesting that the MFC/ACC deficits in inhibitory fMRI meta-analyses (Hart et al., 2013; Norman et al., 2016) may be due to the Go/no-go rather than the Stop task. Another fMRI meta-analysis that included 16 Go/no-Go and 8 Stop task fMRI studies confirmed the findings of underactivation previously observed in left ACC/SMA, bilateral DLPFC, right caudate, left thalamus and left IFC (Lei et al., 2015). However, unlike the other previous three meta-analyses (Hart et al., 2013; McCarthy et al., 2014; Norman et al., 2016), they also found enhanced activation in ADHD in bilateral ventrolateral prefrontal cortex, right precentral and occipital cortices (Lei et al., 2015). A recent individual study in a large number of 185 ADHD patients showed reduced activation in left IFC and superior frontal cortex, ACC and bilateral temporo-parietal regions, which furthermore correlated in left IFC with ADHD symptom severity and stop task performance. The deficits were also present, albeit to a lesser degree, in unaffected siblings suggesting that the fronto-parieto-temporal dysfunction during inhibitory control is familial (van Rooij et al., 2015). In conclusion, meta-analyses of fMRI studies of inhibitory control find most consistently reduced activation in right, left or bilateral IFC, MFC/ACC/SMA and striato-thalamic regions with some studies also finding DLPFC underactivation (Hart et al., 2013; McCarthy et al., 2014; Lei et al., 2015; Norman et al., 2016).

A meta-analysis of a relatively wide range of attention tasks such as selective, divided and sustained attention, as well as alerting and mental rotation included 13 mostly pediatric whole-brain fMRI studies and found reduced activation in 171 ADHD patients relative to 178 healthy controls in the right hemispheric dorsal attention network, comprising right DLPFC, right inferior parietal cortex and caudal parts of the basal ganglia and thalamus. In addition, ADHD patients had increased activation relative to controls in right cerebellum and left cuneus, presumably compensating for the reduced activation of the frontal part of the dorsal DLPFC-parieto-cerebellar attention network (Hart et al., 2013; see Figure 1D). A meta-analysis of timing functions in ADHD, including 11 fMRI studies of time discrimination, time estimation, motor timing and temporal discounting (temporal foresight), showed consistently reduced activation in 150 ADHD patients relative to 145 healthy controls in left IFC, left inferior parietal lobe and right lateral cerebellum (Hart et al., 2012), all key regions of timing functions (Wiener et al., 2010; see Figure 1E). Interestingly, the functional deficits during timing tasks were predominantly left-hemispheric, while the dysfunctions during attention and inhibition functions were predominantly right-hemispheric, in line with a more prominent role of the right hemisphere for attention and inhibition functions (Corbetta et al., 2008; Chambers et al., 2009), while timing functions seem to be mediated by bilateral frontal, insular and striatal regions (Wiener et al., 2010).

A meta-analysis of N-back WM fMRI studies showed that 111 ADHD patients relative to 113 controls had reduced activation in bilateral middle and superior PFC and left MFC/ACC (McCarthy et al., 2014). A recent, relatively large numbered study in over 100 ADHD children and adults using a visual-spatial WM task, however, found a dissociated effect depending on WM load, with enhanced activation in IFC pars opercularis under high memory load, but reduced activation in the triangular part of the IFC during low WM load (Van Ewijk et al., 2015). Last, an older large meta-analysis that included 55 whole-brain fMRI studies of a range of EF, attention, reward and emotion processing tasks in 16 adult and 39 pediatric studies, found reduced activation in 741 ADHD patients relative to 801 controls in different functional brain systems, including the bilateral ventral attention system (IFC, basal ganglia) and predominantly right hemispheric fronto-temporo-parietal cognitive control networks, including DLPFC/IFC, basal ganglia, thalamus, ACC and SMA (Cortese et al., 2012), which overlap with the findings of the above reviewed task-domain specific meta-analyses (Hart et al., 2012, 2013; McCarthy et al., 2014; Lei et al., 2015; Norman et al., 2016).

It is possible that these functional abnormalities express a delay in functional brain maturation. This would be supported by indirect evidence that the reduced regional activations in ADHD patients relative to their age-matched peers during inhibition (Hart et al., 2013; McCarthy et al., 2014; Lei et al., 2015; Norman et al., 2016), attention (Hart et al., 2013), WM (McCarthy et al., 2014) and timing functions (Hart et al., 2012) are in brain regions that have shown to increase in activation progressively between childhood and adulthood during the same motor response inhibition (i.e., IFC, basal ganglia, ACC and SMA; Rubia et al., 2006, 2007, 2013), sustained attention (i.e., DLPFC, parietal lobe and basal ganglia), WM (DLPFC; Klingberg et al., 2002) and timing tasks (i.e., left IFC; Smith et al., 2011; for review see: Rubia, 2013), suggesting that the activation pattern in ADHD patients is like that of younger relative to older children. A delay in brain function maturation would parallel evidence for a maturational delay in brain structure (Shaw et al., 2007, 2012) and in functional connectivity (Sripada et al., 2014b). Longitudinal fMRI studies, however, are needed to corroborate this hypothesis. The findings of domain-dissociated deficits in distinct IFC/ACC/SMA fronto-striato-thalamic (inhibition), right DLPFC fronto-striato-thalamo-parietal (for attention), bilateral DLPFC and MFC/ACC (WM), and left IFC-parieto-cerebellar networks (timing) in ADHD patients for these different cognitive domains suggest that ADHD patients suffer from multisystem deficits compromising different fronto-striato-parieto-cerebellar networks that mediate several cognitive domains (Rubia et al., 2014a).

**Figure 2:**
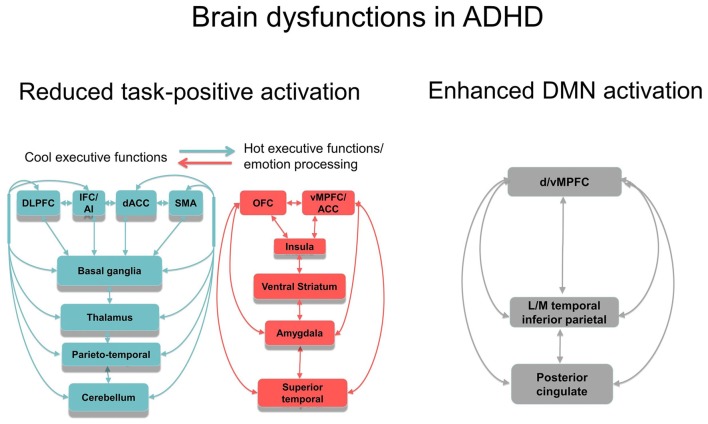
Schematic representation of the most consistent brain function abnormality findings in ADHD. Reduced function and functional connectivity have been observed in several dorsal, ventral and medial fronto-striato-thalamo-parietal and fronto-striato-thalamo-cerebellar networks for cool executive functions (EFs), depending on the task domain tested, including working memory (WM), inhibition, attention and timing. There is emerging evidence for abnormal function and interregional functional connectivity in hot EF networks, most prominently in ventral striatum, but also in lateral and medial OFC and vMPFC, insula, amygdala and superior temporal regions. Furthermore, there is evidence for abnormally reduced deactivation in anterior and posterior regions of the DMN during cognitive tasks. The poor within network connectivity in task-relevant and DMN regions as well as the poor anti-correlation between both appears to be associated with a maturational lag. Both, reduced task-positive activation and reduced deactivation of the DMN is likely to underlie poor cool and hot executive functioning in ADHD. Abbreviations: DLPFC, dorsolateral prefrontal cortex; IFC, inferior frontal cortex; dACC, dorsal anterior cingulate cortex; SMA, supplementary motor area; OFC, orbitofrontal cortex; vMPFC/ACC, ventromedial prefrontal cortex/anterior cingulate cortex; LM, lateral/medial.

Not only task-relevant regions, however, seem to be reduced in function in ADHD. Several of the above reviewed meta-analyses also report increased activation in ADHD patients in regions of the default mode network (DMN). Thus, ADHD patients showed enhanced activation in typical regions of the DMN such as in rostromedial prefrontal cortex during interference inhibition (Hart et al., 2013; Figure 1B), and in posterior cingulate and precuneus (PPC) during motor inhibition, attention (Hart et al., 2013) and timing tasks (Hart et al., 2012; Figures 1A,D,E). Enhanced activation was also observed predominantly in DMN occipital regions in the meta-analysis of 55 fMRI tasks including cognitive control, emotion processing and reward tasks (Cortese et al., 2012) and in a meta-analysis of motor response inhibition (Lei et al., 2015). These findings of enhanced activation of DMN regions confirm recent evidence that poor deactivation of the DMN during cognitive tasks is a typical feature of ADHD and may contribute to poor task performance and enhanced distractibility (Fassbender et al., 2009; Christakou et al., 2013; Salavert et al., 2015). Thus, in parametric fMRI task designs, ADHD children and adults, unlike controls, do not progressively deactivate anterior (ventromedial prefrontal cortex, vmPFC) and/or posterior default mode regions (PPC) with increasing WM or attention load, respectively (Fassbender et al., 2009; Christakou et al., 2013; Salavert et al., 2015). Furthermore, the poor deactivation of DMN regions was inversely associated with worse performance and decreased fronto-striatal activation (Christakou et al., 2013). Both, cognitive control networks and the DMN, develop functionally progressively with age (Sato et al., 2014) and problems to deactivate the DMN during cognitive functions have been associated with more attention lapses, both in normal development and in ADHD (Broyd et al., 2009; Fassbender et al., 2009; Christakou et al., 2013). A recent connectomic study of a large multi-site resting state dataset (ADHD200) in 7–21 year olds, found in fact an age by ADHD severity interaction in 133 ADHD patients relative to 288 healthy controls, suggesting that ADHD patients have a maturational lag in the connectivity *within* the ventral attention, the fronto-parietal cognitive control networks and the DMN, as well as in the negative anti-correlation *between* these task-positive networks and the DMN (Sripada et al., 2014a). The findings overall suggest that ADHD patients have less control over their interoceptive attention orientation and mind-wandering which intrudes more into their already weak exteroceptive attention processes, likely causing enhanced inattention and impulsiveness. This immature pattern of poor activation of task-relevant and age-correlated task-positive brain activation networks and of decreased deactivation of the DMN are likely underlying the poor performance in ADHD on attention-demanding higher-level cognitive control tasks (Rubia et al., 2014a; see Figure 2).

## fMRI Studies of Hot EF and Emotion Processing Tasks

In addition to deficits in several lateral fronto-striato-parietal and fronto-cerebellar regions that mediate so-called “cool” EF, ADHD children have also shown reduced activation in ventromedial prefrontal cortex (vmPFC) or orbitofrontal cortex (OFC) and striato-limbic regions during tasks that tap into “hot” EF such as reward-related decision making or temporal discounting tasks. One of the most consistent findings is reduced ventral striatum activation during reward anticipation, as shown in a recent meta-analysis of eight fMRI studies of a monetary reward anticipation task using region of interest analysis in 340 ADHD patients and healthy controls (Plichta and Scheres, 2014). However, while reward anticipation is associated with diminished ventral striatum activity, presumably due to diminished temporal foresight or predictive dopamine signaling, the reward delivery itself has been shown to be associated with increased activity in reward regions such as ventral and dorsal striatum in young adults with ADHD (Furukawa et al., 2014) and in ventral striatum and superior frontal motor regions and their connectivity in adolescents with ADHD during a rewarded Stroop interference inhibition task (Ma et al., 2016).

With respect to the ventromedial and orbitofrontal parts of the reward processing networks, findings have been more inconsistent. Some studies found abnormally enhanced (Ströhle et al., 2008; Rubia et al., 2009b), others abnormally reduced OFC activation during reward delivery (Dibbets et al., 2009; Rubia et al., 2009a; Cubillo et al., 2012). In response to monetary loss, one study found no differences in adults with childhood ADHD (Stoy et al., 2011), while another found enhanced activation in 28 ADHD adults in temporal pole and limbic regions of amygdala and insula (Wilbertz et al., 2017). Interestingly, the anticipation of forced waiting times, which are particularly aversive to individuals with ADHD (Sonuga-Barke et al., 2010) also elicited enhanced amygdala activation (Wilbertz et al., 2013).

Few studies have measured brain response to delay discounting tasks, which are impaired in ADHD, with, however, also some negative findings (Noreika et al., 2013). Delay discounting tasks measure both “cool” and “hot” EFs such as motivation control, delay aversion and temporal foresight (Noreika et al., 2013). Reduced activation and abnormal brain-behavior correlations during temporal discounting have been observed in ADHD children and adults most prominently in typical areas of temporal discounting including ventrolateral and dorsolateral prefrontal cortices, insular, dorsal and ventral striatal and thalamic regions as well as parietal lobe and cerebellum (Rubia et al., 2009a; Chantiluke et al., 2014; Ortiz et al., 2015; Carlisi et al., 2016; Norman et al., 2017a), while one study found additional enhanced activation in adults with ADHD in dorsal caudate and amygdala to delayed rewards (Plichta et al., 2009).

More recently, evidence has emerged that ADHD patients have also emotional dysfunctions, most prominently problems with emotion regulation, which has been argued to be related to poor top-down executive control over enhanced bottom-up emotional reactivity, resulting in enhanced disinhibitory and aggressive behaviors (Barkley and Fischer, 2010; Graziano et al., 2013; Shaw et al., 2014). Thus, during emotional distractors in the emotional Stroop and a WM task, two fMRI studies found reduced activation in ADHD patients during negative valenced stimuli in ventral and mPFC, subgenual ACC, striatum and temporo-parietal regions (Passarotti et al., 2010; Posner et al., 2011a), but greater activation in dorsolateral prefrontal cortex (DLPFC; Passarotti et al., 2010), in left temporal and occipital (Hwang et al., 2015) and in vmPFC/subgenual ACC, striatum, and temporo-parietal regions (Passarotti et al., 2010; Posner et al., 2011a) during positive emotional distractors. During the processing of fearful facial expressions, children and adults with ADHD compared to healthy controls showed enhanced activation in the amygdala (Brotman et al., 2010; Posner et al., 2011b) and its functional connectivity with PFC (Posner et al., 2011b). These findings are in line with the above mentioned findings of enhanced amygdala, insula and temporal pole activation in ADHD during monetary loss (Wilbertz et al., 2013, 2017) and suggest that ADHD patients have exaggerated neuro-functional responses to negative (and positive) emotional and reward stimuli. However, other studies found no differences in ADHD children in brain activation to fearful faces (Marsh et al., 2008) or negative emotional pictures (Herpertz et al., 2008) or decreased amygdala activation in children but enhanced activation in adults with ADHD during fearful and angry faces (Bottelier et al., 2017) and reduced activation to verbally instructed fear-conditioning in subgenual ACC (Maier et al., 2014) and in ventral striatum and subgenual ACC in response to unexpected negative and positive pictures (Schlochtermeier et al., 2011). Also, during reactive aggressive responses, ADHD adolescents showed abnormally reduced activation in ACC and temporo-parietal junction as well as in striato-limbic regions (Bubenzer-Busch et al., 2016). A recent study found abnormal activation in fronto-limbic regions during extinction learning and recall in adults with ADHD including hippocampus, insula (overactivated) and mACC and vmPFC (underactivated), suggesting medial frontal-limbic dysregulation similar to that observed in post-traumatic stress disorder (Spencer et al., 2017).

In conclusion, the findings of brain abnormalities in ADHD during reward and emotion processing are relatively inconsistent, with some studies finding neuro-functional hyper-responsiveness in OFC/vmPFC–limbic regions to negative and positive emotions, but this has not been confirmed in other studies. These inconsistent findings are likely due to small sample sizes, confounds of previous medication history, and the presence of comorbidities, in particular CD and ODD which have been associated with ventromedial and dorsomedial-limbic dysfunctions during hot EF and emotion processing (Rubia, 2011; Alegria et al., 2016). Another important caveat is that dysfunctions in ventral striatum and amygdala have almost exclusively been observed in region of interest studies. Future larger numbered whole-brain fMRI studies in well-defined, medication-naïve and non-comorbid ADHD populations will have to confirm the presence of abnormalities in these ventromedial and orbital frontal limbic-ventral striatal systems (see Figure 2).

## Task-Based Functional Connectivity Deficits

Relatively few fMRI studies have tested for abnormalities in functional connectivity during cognitive tasks using either seed-based task-specific correlations of predefined regions of interest and independent component methods or effective connectivity methods (i.e., psycho-physiological interaction, structural equation modeling and Granger causal modeling), which are hypothesis-driven and measure changes in interactions across brain activations. In children and adults with ADHD, reduced functional connectivity has been observed between task-relevant regions during cool EF tasks, suggesting dysfunction of entire networks and not just regions.

In ADHD children, during motor response inhibition and WM tasks reduced functional connectivity has been reported relative to healthy controls between the right IFC and basal ganglia, parietal lobes and cerebellum, and between cerebellum, parietal and striatal brain regions during sustained attention (Rubia et al., 2009b), interference inhibition and time estimation (Vloet et al., 2010). Some studies have in addition found increased activation in fronto-parietal and auditory networks (Wu et al., 2017). During the Stroop task, left dorsomedial prefrontal cortex showed reduced functional connectivity with right lateral prefrontal cortex, but increased connectivity with left insula (Hwang et al., 2015).

In adults with ADHD, reduced functional connectivity relative to healthy controls was observed between bilateral IFC, and between right IFC and striatal, cingulate, parieto-temporal and cerebellar regions during motor response inhibition and WM tasks (Wolf et al., 2009; Cubillo et al., 2010). A reduction of thalamo-cortical connectivity was also observed during a simpler task of response preparation (Clerkin et al., 2013). In adults, however, there is also additional evidence for increased connectivity during WM between ACC, superior frontal lobe and cerebellum, presumably compensatory (Wolf et al., 2009).

During emotion processing, abnormally enhanced functional connectivity has been observed between limbic and orbitofrontal regions. Thus, adults with ADHD showed enhanced functional connectivity between amygdala and left lateral prefrontal cortex during negative emotions (Posner et al., 2011b). Similarly, happy distractors in the emotional Stroop task elicited enhanced connectivity in ADHD patients between the amygdala and striatal and occipital regions (Hwang et al., 2015). The findings suggest emotional hyper-responsivity to positive and negative emotions.

As mentioned above, a large multi-site resting state fMRI connectomic study found that ADHD patients relative to healthy controls have a maturational lag in the connectivity *within* task-positive networks such as the ventral and dorsal attention networks and the DMN, as well as in the interaction *between* these task-positive networks and the DMN (Sripada et al., 2014a).

To summarize, task-based functional connectivity studies suggest that abnormalities in brain function in ADHD children and adults is associated with a disturbance in wide-spread task-based functional neural networks, observed both at rest and during cognitive and emotion functions with evidence that in resting state fMRI data this may be associated with a maturational lag. Abnormal task-based functional connectivity is likely also due to a delay in functional maturation, given that task-based functional connectivity increases progressively with age (Rubia, 2013), but this will need to be corroborated in longitudinal fMRI studies.

## ADHD Subtypes

Little is known on the neuro-functional differentiation of ADHD subtypes. The first fMRI study to compare ADHD subtypes found that children with the inattentive only ADD subtype had larger activation in middle frontal, temporal and parietal regions, whereas children with ADHD-hyperactive/impulsive and inattentive combined type activated bilateral medial occipital lobe to a greater extent than children with the inattentive subtype (Solanto et al., 2007). One of the fMRI meta-analyses compared ADHD subtypes and showed that combined-type ADHD relative to the inattentive subtype had more severe underactivation in right superior and IFC during the Stop task, in right caudate during the Go/no-go task and in right cerebellum during the WM task. In addition, areas of the DMN such as medial frontal and occipital regions were more enhanced in activation in the combined type ADHD subgroup relative to controls for all tasks (McCarthy et al., 2014).

## Impact of Conduct and Oppositional Defiant Disorder

The majority of fMRI studies in ADHD have excluded the presence of comorbid major psychiatric conditions, with the exception of CD and ODD, as they are highly prevalent in the disorder, with between 60%–78% of comorbidity for ODD (Costello et al., 2003; Connor et al., 2010) and up to 50% for CD (Maughan et al., 2004). Consequently, it is difficult to ascertain whether the brain dysfunctions associated with ADHD are truly due to ADHD or to the comorbid presence of CD/ODD. Several of the fMRI meta-analyses, however, found no association between brain dysfunctions and comorbid conditions (Cortese et al., 2012; Hart et al., 2012).

The very few comparative fMRI studies that compared small numbers of non-comorbid ADHD and non-comorbid CD/ODD children showed that ADHD is associated with DLPFC and IFC underactivation in four out of five fMRI tasks, while CD/ODD was associated with paralimbic underactivation in orbitofrontal, limbic and superior temporal regions (Rubia et al., 2008, 2009c,d, 2010b), for review see Rubia (2011). Two fMRI studies compared ADHD children with and without CD and psychopathy traits, and found that only the comorbid group had reduced amygdala activation and reduced functional connectivity between amygdala and vmPFC in relation to fear (Marsh et al., 2008), but enhanced activation in vmPFC during punished reversal errors, both of which correlated with their antisocial and psychopathy traits (Finger et al., 2008). A recent meta-analysis of fMRI studies of cool and hot EF as well as emotion processing in CD/ODD showed that the most consistent underactivation was in ventral and dorsal medial prefrontal cortex, which was mostly triggered by hot EF tasks (Alegria et al., 2016). In conclusion, it appears that there are disorder-specific and process-related dissociations in prefrontal functional deficits between both disorders, with ADHD children having consistent problems with the recruitment of lateral IFC/DLPFC systems in the context of “cool” executive inhibitory and attention control, whereas CD children have problems with the recruitment of “hot” vmPFC and OFC-limbic systems that mediate top-down control over motivation and affect (Rubia, 2011; Alegria et al., 2016). The comorbid presentation is likely to suffer from a dysregulation of both “cool” fronto-striato-parieto-cerebellar as well as “hot” ventromedial fronto-temporo-limbic neural networks. However, this will need to be tested in future large-numbered fMRI studies that compare brain activation deficits in patients with non-comorbid and with comorbid conditions to disentangle disorder-specific and shared brain function abnormalities.

## Persistence of fMRI Deficits Into Adult ADHD

For fMRI studies to be comparable it is crucial that the same fMRI paradigms are used. Cross-sectional fMRI studies that used identical cool and hot EF tasks in children and adults with ADHD suggest that adults with ADHD, if they persist with their ADHD symptoms, have similar brain activation deficits as children with ADHD (for review, see Cubillo and Rubia, 2010; Cubillo et al., 2012). Similarly, most of the fMRI meta-analyses found no significant linear or categorical age effects (Cortese et al., 2012; Hart et al., 2012, 2013). However, most meta-analyses included only small numbers of adult fMRI studies. A categorical age comparison for a meta-analysis of fMRI studies on motor and interference inhibition, where more adult fMRI studies were available, showed that ADHD children had more pronounced SMA/ACC and basal ganglia dysfunctions, while ADHD adults had more pronounced inferior frontal and thalamus dysfunctions (Hart et al., 2013). This was replicated in two subsequent larger fMRI meta-analyses of cognitive control (Norman et al., 2016; Rubia et al., 2016). Another meta-analysis of fMRI studies of motor inhibition found that children had significantly more underactivation in right caudate than adults with ADHD (Lei et al., 2015). Right fronto-parietal underactivation was observed in an adult sub-meta-analysis across 55 fMRI tasks of different functions, although no differences were observed between pediatric and adult samples (Cortese et al., 2012). Two recent studies comparing persisters and non-persisters in fMRI during motor and interference inhibition tasks found that while caudate underactivation was associated with childhood ADHD (Szekely et al., 2017), only the ADHD persisters in adulthood had underactivation in ventrolateral prefrontal cortex, parietal regions (Schulz et al., 2017; Szekely et al., 2017), anterior cingulate (Schulz et al., 2017) and cerebellum (Szekely et al., 2017), while non-persisters did not differ from controls (Schulz et al., 2017; Szekely et al., 2017). The findings of fronto-parietal dysfunction- which mediates attention- in adult ADHD persisters is in line with evidence that attention deficits typically persist in adult ADHD and with evidence for abnormal cortical thinning in lateral fronto-parietal regions in ADHD persisters, but not remitters (Shaw et al., 2013). The recent, large-numbered study in 185 ADHD subjects, however, found no age effects for inhibition dysfunctions (van Rooij et al., 2015). A more conclusive test of persistence of fMRI deficits is in longitudinal studies. However, only one longitudinal fMRI study exists that found that children with ADHD as well as the re-assessed remitting and persisting adults with ADHD had reduced activation in MPFC and striatum during punished reward reversal (Wetterling et al., 2015). However, only remitters and controls presented significant psycho-physiological interaction between these fronto-striatal reward and outcome valence networks and there was evidence for compensatory activation in left prefrontal regions in the remitters (Wetterling et al., 2015).

In conclusion, there is thus evidence that basal ganglia deficits may be more pronounced in ADHD children while fronto-cortical dysfunctions appear to persist or become even stronger in adult ADHD persisters. The fMRI findings parallel evidence from structural MRI studies for more abnormal basal ganglia deficits in childhood than adult ADHD (Norman et al., 2016; Rubia, 2016; Hoogman et al., 2017). Future meta-analyses in larger numbers of adult fMRI studies are needed to confirm that adults and children with ADHD suffer from similar activation deficits. More importantly, more longitudinal fMRI studies following up ADHD persisters and remitters from childhood ADHD will be crucial to define the neurofunctional developmental trajectories of persisting and remitting ADHD.

## Gender Effects

ADHD is more prevalent in males, in particular in childhood. Therefore, relatively few fMRI studies have tested females or sex differences in fMRI activation. A study in adults with ADHD found that 23 males had significant underactivation in widespread networks of frontal, temporal, cerebellar, occipital and subcortical regions during WM, whereas 21 females with ADHD showed no impairment relative to control females (Valera et al., 2010). The findings are in line with another study including only female ADHD adolescents that also found no differences in WM-related brain activation (Sheridan et al., 2007). A small study comparing 23 ADHD with 21 healthy adolescents during assessment of congruent or incongruent stories, found that ADHD males had bilateral frontal and parietal underactivation compared to controls together with hyperactivation of amygdala and superior temporal motivation regions, while ADHD females had a more widespread underactivation pattern in right inferior frontal and postcentral gyri, right culmen, right middle temporal gyrus and left basal ganglia. However, the study was severely underpowered with only seven girls (Poissant et al., 2016). By contrast, a recent well-powered fMRI study using the Stop task in 185 patients with ADHD, found no sex differences (56 females) on the underactivation of IFC, DLPFC, ACC and temporo-parietal regions (van Rooij et al., 2015). The largest fMRI meta-analysis that included a range of cool, hot EF and emotion processing tasks, also found no sex differences in activation deficits (Cortese et al., 2012). However, females are underrepresented in fMRI studies due to the increased prevalence of male ADHD and larger-numbered fMRI studies including equal gender proportions are needed to confirm whether there are sex differences in functional brain activation in ADHD.

## Disorder-Specificity of Deficits

Finding disorder-specific neurofunctional biomarkers for ADHD is particularly important to aid with a more objective differential diagnosis or differential treatment approaches. IFC underactivation has been found to be disorder-specific to ADHD in the context of cognitive control functions relative to other childhood disorders. Thus, during cognitive control tasks, IFC was found to be disorder-specifically reduced compared to OCD patients in individual fMRI studies (Rubia et al., 2010a, 2011a), as well as in a comparative fMRI meta-analysis of cognitive control functions, including motor and interference inhibition and switching tasks, in 541 ADHD and 287 OCD patients (Norman et al., 2016). IFC underactivation was also found to be disorder-specific in individual fMRI studies compared to ASD (Chantiluke et al., 2015) and in a comparative meta-analysis of cognitive control in 629 ADHD relative to 208 patients with ASD (Rubia et al., 2016). IFC underactivation during cognitive and motivation control tasks was also found to be disorder-specific to ADHD relative to borderline personality (Sebastian et al., 2014) and bipolar disorder (Passarotti and Pavuluri, 2011). These more “affective childhood disorders” appear to suffer from similar underactivations in ventromedial and/or dorsomedial prefrontal and limbic regions during cognitive control (Passarotti and Pavuluri, 2011; Sebastian et al., 2014; Alegria et al., 2016; Norman et al., 2016; Rubia et al., 2016). However, in other task domains the disorder-specificity of ADHD dysfunctions may be less pronounced. For example, during attention tasks, common deficits in attention networks were found in ADHD, OCD, and ASD, although dorsolateral prefrontal dysfunction was more pronounced in ADHD compared to both ASD and OCD (Christakou et al., 2013; Norman et al., 2017b). Similarly, during hot EF such as temporal discounting tasks, brain dysfunctions were shared between ADHD and OCD in ventromedial prefrontal and striatal regions (Norman et al., 2017a) and mostly shared between ADHD and ASD in inferior frontal cortex and SMA (Chantiluke et al., 2014). The comorbid ADHD and ASD group had the most neurofunctional abnormalities in regions mediating temporal discounting, including ventrolateral and dorsolateral prefrontal, cingulate, striato-insular, temporal and cerebellar regions (Chantiluke et al., 2014). In response to monetary reward, shared reductions were found between ADHD and ASD in reward and salience processing regions including the dorsal striatum, anterior and posterior cingulate, thalamus, while ADHD patients had disorder-specific enhanced dorsal ACC and superior frontal activation in response to social rewards (Kohls et al., 2014).

The findings thus suggest that disorder-specific abnormalities in ADHD may be context-dependent with more distinctive abnormalities during cognitive control than hot EF or attention tasks. However, this will have to be confirmed in future comparative meta-analyses of fMRI studies across different cognitive domains and disorders.

## Translational Cognitive Neuroscience of ADHD

Clinical translation of neuroimaging is still in its childhood and will be the challenge over the next decades. For neuroimaging to have clinical use, it will have to help with providing clinical diagnosis, prognosis or treatment. Several studies have used multivariate pattern recognition analyses in an attempt to provide diagnostic classification of ADHD patients relative to controls based on task-based fMRI neuroimaging data, with relatively high classification accuracy. The establishment of neurofunctional biomarkers for ADHD with fMRI studies has made it possible to target these biomarkers using therapeutical neuroimaging. Thus, NF therapies using real-time fMRI or NIRS using these neurofunctional biomarkers as treatment targets have recently been applied to ADHD children and adults with somewhat promising results. Other non-invasive neurotherapies such as regional magnetic or electrical stimulation using repetitive transcranial magnetic stimulation (rTMS) and transcranial direct current stimulation (tDCS) have found successful applications in other psychiatric disorders. Pioneering applications of these techniques to ADHD over the past decade targeting IFC or DLPFC have been mixed, but revealed some promising findings of improving cognition and clinical behavior. The following sections will review these clinical applications of neuroimaging in ADHD.

## Pattern Recognition Analyses of fMRI Data for Diagnostic Classification of ADHD Patients

Despite the fact that ADHD is a neurodevelopmental disorder with consistent evidence for brain structure and function deficits, currently ADHD is diagnosed solely on the basis of subjective clinical and self-rating measures, which are often unreliable, leading to diagnostic variability between clinicians, cultures and countries (Polanczyk et al., 2007). Sensitivity of classification of ADHD children with clinical measures based on DSM-IV criteria, which is the gold-standard behavioral measure for ADHD diagnosis, has been shown to be between 70%–90% (Weiler et al., 2000), thus misdiagnoses are around 10%–30%. It is thus highly desirable to develop additional and more reliable diagnostic methods for ADHD patients based on objectively measurable neuroimaging data.

Multivariate pattern analyses for imaging data take into account interactions between regions (i.e., brain structure or function patterns) and can make predictions (e.g., of class membership) for individual subjects as opposed to group-level inferences. These methods have been shown to provide sensitive and specific diagnostic indicators for individual patients with other pathologies in particular for neurological disorders such as Alzheimer’s disease but also for autism and depression (for review, see Orrù et al., 2012; Wolfers et al., 2015).

Several pioneering machine learning approaches applied to fMRI data have been promising, showing relatively high accuracy of up to 80% in classifying ADHD patients relative to controls. Two fMRI studies using Gaussian processes in fMRI data in adolescents with ADHD showed a relatively high overall classification accuracy of almost 80% with relatively small numbers of about 30 patients for inhibition and timing functions (Hart et al., 2014a,b). Another study classified 78% of ADHD adolescents based on error processing fMRI data during an interference inhibition/Go/no-go task (Iannaccone et al., 2015). A more recent study used a logistic regression classifier on fMRI data from four distinct visual-spatial WM tasks that modulated reward and feedback in ADHD and healthy boys. The study used a multimodal logistic regression classifier based on brain activity in 16 regions of interest, significantly activated or deactivated in the four visual-spatial WM tasks (based on the entire participants’ sample) and enabled a classification accuracy of 92.5%, with high predicted ADHD probability values for most clinical cases, and low predicted ADHD probabilities for most healthy controls. This accuracy level was higher than those achieved by using the fMRI data of any single task or the behavioral data (Hammer et al., 2015). A recent study used support vector machine classification to predict the diagnosis of relatively small numbers of 13 adult ADHD subtypes of inattentive and 21 impulsive-hyperactive and inattentive combined type ADHD based on connectivity differences in six fMRI tasks. Classifier accuracy for distinguishing between ADHD subtypes was 91.18% for a gambling punishment and emotion task paradigms based on significant connectivity differences mainly in frontal, cingulate, and parietal cortices and partially in temporal, occipital cortices and cerebellum (Park et al., 2016).

These multivariate classification approaches using functional imaging data seem promising. However, analyses were based on small and largely homogenous samples and generalisability is questionable. Structural and resting state functional connectivity data have been more commonly used in ADHD due to their larger comparability across centers allowing for multi-site analyses. The ADHD Consortium, 2012, a data-sharing project, called a competition for different groups from all over the world to train their machine-learning algorithms on a multi-site dataset on about 350 ADHD patients and 554 controls on data including demographic, clinical, structural and resting-state fMRI data. The classification results were very low with accuracies not exceeding 61% on the test sample and clinical data were more predictive of ADHD classification than imaging data (Wolfers et al., 2015). Also, discriminative classification of more than one disorder to aid with differential diagnosis may be more useful. So far, however, only one pattern recognition analysis study used structural imaging data to differentially classify 44 ADHD patients compared to 19 patients with autism and 33 healthy controls, and achieved a relatively high classification accuracy of over 90% (Lim et al., 2013).

Whilst imaging-based classification algorithms are unlikely to replace clinical assessment and diagnosis, they may be a useful objective, automated, and reliable screening method or a complementary diagnostic tool that could reduce variability in clinical practice and, ultimately, help to improve diagnostic accuracy or revise clinical diagnosis through biomarker classification of uncertain diagnostic cases. Furthermore, these methods may be more useful for prognostic rather than diagnostic classification, such as predicting the disease progression, adult outcome of ADHD or medication response, given that brain mechanisms are likely to be better predictors of disease progression or medication response than behavioral measures. There is hence a potential that these methods could improve clinical practice and personalized medicine. However, they need to show replicability across different representative patient groups, scanners and demographic populations, before they can be used to help with future imaging-based (differential) diagnosis or prognosis of individual patients and build the path for brain function (or brain structure)-based patient stratification and personalized medicine. Multimodal multivariate approaches including several imaging modalities, including functional and structural imaging data as well as non-imaging data such as cognitive and genetic measures are likely to achieve superior classification accuracy than univariate approaches (Orrù et al., 2012; Wolfers et al., 2015). The high etiological and phenotypic heterogeneity that characterizes ADHD makes classification and its generalizability difficult. Methodological innovations are needed to improve accuracy and to discriminate between multiple disorders simultaneously (Wolfers et al., 2015). The combination of technological developments in pattern recognition methods with the acquisition of large, multimodal clinical samples will hopefully allow more accurate disorder classification and move the field closer towards biomarkers that can assist with clinical decision making (Wolfers et al., 2015).

## Brain Stimulation

The last decade of neuroimaging has shown that the brain is highly plastic, in particular in childhood/adolescence, when it is still developing (Rapoport and Gogtay, 2008; Jäncke, 2009). This makes novel treatments using transcranial neuromodulation an attractive clinical intervention (Ashkan et al., 2013), in particular at early stages of the disorder, in young people, where it is likely to be most effective (Anderson et al., 2011). In fact, children and adolescents show accelerated neural plasticity compared to adults after brain stimulation (Brunoni et al., 2012). Non-invasive brain stimulation therapies, most prominently rTMS and tDCS, have only over the past years been applied to ADHD. These stimulation techniques affect cellular and molecular mechanisms involved in use-dependent local and distant synaptic plasticity, i.e., GABA and glutamate-mediated long-term potentiation, which may lead to longer-term effects (Demirtas-Tatlidede et al., 2013).

## Repetitive Transcranial Magnetic Stimulation (rTMS)

rTMS is a non-invasive and safe brain stimulation technique that uses brief, intense pulses of electric current delivered to a coil placed on the subject’s head in order to generate an electric field in the brain via electromagnetic induction. A commonly used figure-8 coil provides relatively focal stimulation of approximately 5 mm^3^. The induced electrical current triggers action potentials in the brain via current flowing parallel to the surface of the coil and thus modulates the neural transmembrane potentials and therefore neural activity. The magnitude of the stimulation is inversely related to the distance from the coil. The effect differs depending on the intensity, frequency, and number of pulses applied; the duration of the course and the coil location. In general, high-frequency (>5 Hz) rTMS promotes cortical excitability, while low frequency (1 Hz) rTMS inhibits cortical excitability (Lefaucheur et al., 2014).

Three studies have applied rTMS to adults with ADHD so far. A study by Bloch et al. (2010) tested rTMS over the right DLPFC in 13 adult ADHD patients applying 42 2 s 20 Hz stimulations interspersed with a 30 s inter-stimulus interval in the real TMS condition and no stimulation in the sham stimulation condition in a cross-over trial. There was a group by treatment interaction with only the group that received real rTMS stimulation improving in behavioral attention measured 10 min after the stimulation (but not in mood and anxiety scores or in cognitive measures). A second crossover study applied rTMS over right prefrontal cortex in 9 young adults with ADHD. rTMS stimulation was set at 100% the motor threshold, 10 Hz, with 2000 pulses per day over 10 treatment days. Clinical improvement were shown in both the sham and the real rTMS groups, suggesting placebo effects, while no effects were observed for neuropsychological or EEG measures (Weaver et al., 2012). A third, larger study randomized 22 ADHD adults into deep TMS (dTMS; *N* = 9) or sham dTMS (*N* = 13). dTMS is a modification of standard TMS that enables deeper non-invasive cortical stimulation at an effective depth of approximately 3 cm depending on the coil’s design and the stimulation intensity. Stimulation was applied over bilateral prefrontal cortex in 20 daily sessions over 4 weeks, each session consisting of 55 trains of pulses at 18 Hz (2 s per train, with a 20-s inter-train interval). The study found an improvement in ADHD symptoms in both groups suggesting a placebo effect and no cognitive improvements in either group (Paz et al., 2017). Last, an open-label tolerability and safety study of rTMS of 1 Hz over left DLPFC in 13 children with ADHD study found only temporary minimal side or adverse effects (slight headache) and an improvement in ADHD symptoms, in particular inattention at school and hyperactivity/impulsiveness at home (Gomez et al., 2014). However, the study was not designed to test clinical efficacy as it was open-label.

In conclusion, while the first sham-controlled pilot study using one single session of rTMS in ADHD adults showed positive results, subsequent larger numbered sham-controlled studies reported no superior effects of rTMS over sham rTMS on ADHD symptoms or cognition. Findings are hence not very encouraging. However, a series of studies using the related method of tDCS have been more promising.

## Transcranial Direct Current Stimulation (tDCS)

tDCS is another non-invasive neuromodulation method that applies weak, painless, persistent direct electric currents to specific cortical regions via scalp electrodes with the electrical current passing between a positively charged anode and a negatively charged cathode. In general, currents induce plasticity by facilitating (anodal stimulation) or decreasing (cathodal stimulation) the excitability of neurons via the generation of subthreshold (stimulation-polarity dependent) alterations of membrane potentials that modify spontaneous discharge rates, thus increasing/decreasing cortical function and synaptic strength (Ashkan et al., 2013). tDCS is much easier to apply and has lower financial costs to TMS. Furthermore, tDCS has the advantage of being less painful than TMS and hence is more child-friendly. Side effects are minimal in children (and adults), typically involving transient itching and reddening of the scalp site of stimulation in some participants (Krishnan et al., 2015). Currents are typically applied for up to 30 min, permitting brain stimulation during a cognitive paradigm. tDCS over cortical areas that mediate the cognitive function of interest combined with cognitive training of that specific function can improve task performance (Kuo and Nitsche, 2012), presumably via boosting training-induced plasticity through the addition of stimulation-induced plasticity (Ziemann and Siebner, 2008), yielding larger and long-lasting functional improvements that modify the impaired system (Cramer et al., 2011). This may be helpful in people with deficient neural networks like ADHD patients. tDCS is thought to affect neuroplasticity via modulating cellular, molecular and neurochemical mechanisms involved in use-dependent local and distant synaptic plasticity, i.e., GABA and glutamate-mediated long-term potentiation (Nitsche et al., 2008; Kim et al., 2014), thought to underlie its long-term effects (Demirtas-Tatlidede et al., 2013). Cognitive training effects with tDCS in other disorders and healthy subjects have been shown to last up to 6 months (Boggio et al., 2012; Kuo et al., 2014) and even 1 year (Katz et al., 2017). Functional neuroimaging studies have furthermore demonstrated modulation not only of the site of stimulation but also of functionally interconnected (sub)cortical regions (Polanía et al., 2011), which makes it useful for targeting networks such as fronto-striatal systems in ADHD. Furthermore, relevant to ADHD, prefrontal stimulation has shown to increase striatal dopamine (Pogarell et al., 2007), similar to amphetamines, which is typically reduced in ADHD (Fusar-Poli et al., 2012). Cognitive training alone has shown limited efficacy and transfer effects in ADHD (Cortese et al., 2015). However, it is possible that tDCS combined with cognitive training may be more effective. In healthy adults, for example, tDCS over rIFC combined with inhibition training significantly improved inhibition performance in a Stop task relative to sham tDCS (Cunillera et al., 2014) and to inhibition training alone (Ditye et al., 2012).

In ADHD, to date 10 studies have applied tDCS in ADHD, seven of them using double-blind, two single-blind sham-controlled designs with the only open-label study combining stimulation with cognitive training (see Table 1). Five studies applied tDCS in a single session in children and one in adults with ADHD. The largest study in adult ADHD applied one single session of 20 min of tDCS over left DLPFC with 1 mA in 60 ADHD adults randomized into sham or real tDCS and measured performance in a Go/no-go motor inhibition task, which was not improved by tDCS (Cosmo et al., 2015). Given that the left DLPFC is not a key area of inhibition, it remains to be investigated whether tDCS over right DLPFC or IFC would elicit performance improvement. The findings of a cross-over study in 20 high-school students with ADHD symptoms support this view as they found that a single session of 15 min of cathodal stimulation of 1.5 mA of left DLPFC improved inhibitory performance in a gonogo task, while anodal stimulation of left DLPFC improved the go process of the task, both compared against each other and against sham stimulation. The authors argue that cathodal stimulation of left DLPFC due to interhemispheric inhibition processes may have enhanced right DLPFC activation which is mediating inhibitory performance (Soltaninejad et al., 2015). In fact, a randomized sham-controlled cross-over study combining one session of 15 min of 1.5 mA over right IFC in anodal, cathodal and sham tDCS with performance on a Flanker interference inhibition task found that 21 ADHD adolescents showed significantly reduced commission errors and response variability after the anodal tDCS of rIFC, which was normalized compared to controls (Breitling et al., 2016). However, given that there were learning effects, the analysis focused only on the first session which limited the power of the analysis to seven subjects in each group (Breitling et al., 2016). Similar findings were also made in a third study that tested several combinations of single 15 min sessions of 1 mA tDCS of anodal left DLPFC/cathodal right DLPFC, anodal left DLPFC/cathodal right OFC and of cathodal left DLPFC/anodal right OFC in two randomized, double-blinded sham-controlled studies including groups of 15 and 10 children with ADHD and measured the performance effects on several EF tasks (Nejati et al., 2017). As is to be expected, they found that different regional stimulation protocols benefitted different cognitive tasks. Anodal tDCS of left DLPFC most clearly affected executive control functions (e.g., WM, interference inhibition in the Stroop task), while cathodal left DLPFC/anodal right OFC tDCS improved inhibitory control in a Go/no-go task. Cognitive flexibility benefitted from both anodal left DLPFC/cathodal right OFC and cathodal left DLPFC/anodal right OFC combinations, but not from left anodal DLPFC/cathodal right DLPFC stimulation (Nejati et al., 2017). The findings suggest that tDCS over different prefrontal regions may be necessary to improve the range of cognitive functions that are impaired in ADHD. This would be in line with the fMRI meta-analyses findings suggesting multisystem neurofunctional impairments involving several different lateralised medial, dorsolateral and inferior fronto-striatal networks in ADHD (Hart et al., 2012, 2013; Norman et al., 2016).

**Table 1:** Studies testing the effects of transcranial direct-current stimulation (tDCS) in Attention Deficit Hyperactivity Disorder (ADHD).

Study	Session Nrs	Anodal/cathodal	Region	*N*	Age	Clinical effects	Cognitive effects
Breitling et al. (2016)	1	Anodal/cathodal/sham	rIFC	21	14	n/t	Interference inhibition (Flanker)
Nejati et al. (2017)	1	Anodal/cathodal cathodal/anodal	lDLPFC/rDLPFC	15	10 (2)	n/t	WM (N-back); interference inhibition (Stroop) No effect on GNG, WCST
Nejati et al. (2017)	1	Anodal/cathodal cathodal/anodal	lDLPFC/rOFC	10	9 (2)	n/t	WM, Switching (WCST), Motor inhibition (GNG), switching
Soltaninejad et al. (2015)	1	Anodal/cathodal cathodal/anodal	L DLPFC	20	16 (1)	n/t	Accuracy (GNG) Motor inhibition (GNG) No effect on interference inhibition
Sotnikova et al. (2017)	1	Anodal	L DLPFC/sham	13	14(1)	n/t	RT and SDRT (Qb test: motor inh/WM) Commission and omission errors worse
*Cosmo et al. (2015)	1	Anodal	L DLPFC/sham	60	32 (12)	n/t	No effects (GNG)
Soff et al. (2017)	5	Anodal	L DLPFC/sham	13	14 (1)	Inattention only, after and 7 days later	Hyperactivity mrs (Qb: GNG/WM), also 7 days Inattention mrs (RT, SDRT, OM) at 7 days No effect on impulsiveness (Prem, Com)
Prehn-Kristensen et al. (2014)	5	Anodal	L DLPFC/sham	12 14	12 (1) 12 (1)	n/t	Declarative memory RT and SDRT in motor inhibition (GNG) No effect on alertness and motor memory
Munz et al. (2015)						
*Cachoeira et al. (2017)	5	Anodal/cathodal	lDLPFC/rDLPFC	17	34 (4)	Inattention only, after and 2 weeks later	n/t

DLPFC, dorsolateral prefrontal cortex; COM, commission errors; GNG, Go-no-go task; IFC, inferior frontal cortex; l, left; msrs, measures; OM, omission errors; r, right; RT, reaction time; SDRT, intrasubject standard deviation of reaction time; WM, working memory task; errors; Prem, premature errors. *Studies were in adult ADHD. n/t, not tested.

Two studies tested five repeated sessions of 1–2 mA tDCS of 20 min in double-blind sham-controlled studies on either ADHD symptoms alone (Cachoeira et al., 2017) or additional cognitive functions (Sotnikova et al., 2017). The study in ADHD adults was a randomized double-blind, placebo-controlled clinical trial using sessions of 2 mA stimulation with anodal stimulation of right DLPFC and cathodal stimulation of left DLPFC in 17 adult ADHD patients and found trend-wise significant improvements in overall ADHD symptom scores and a significant improvement in inattention scores after real (*N* = 9) relative to sham stimulation (*N* = 8); furthermore, all symptom improvements were still significant 2 weeks after stimulation, suggesting longer-term effects (Cachoeira et al., 2017). The other study tested five sessions of 1 mA tDCS sessions of 20 min over 5 days in 15 ADHD adolescents in a randomized, double-blinded, sham-controlled crossover study with 2 weeks break between conditions and found significant improvements in inattention symptoms only, which were stronger 7 days later. Furthermore, in cognition, they also found improvements in a WM/sustained attention task of motor activity immediately after stimulation and 7 days later and in attention measures (reaction time and its variability) after 7 days only, but no effects on impulsiveness measures such as commission errors or premature responses (Soff et al., 2017). The fact that behavioral and cognitive effects were stronger 7 days later rather than immediately after the stimulation could potentially suggest longer-term consolidation effects of tDCS on behavior and cognition. Within the same study, they also tested fMRI effects after a single session of tDCS over left DLPFC in 13 of the patients. In the same cognitive WM/attention task, ADHD adolescents improved in reaction time and its variability but became worse in omission and commission errors after stimulation compared to sham (Sotnikova et al., 2017). The findings could suggest a shift in the speed-accuracy trade-off favoring speed, which may be related to the left DLPFC being important for motor initiation. Only one study combined cognitive training in a card matching game with 30 min of 2 mA of tDCS in nine ADHD adolescents in a non-controlled auto-matched open trial. The study found some improvement in switching and visual attention tasks as well as in ADHD symptoms, but without a sham control condition, practice or placebo effects cannot be ruled out (Bandeira et al., 2016). Last, two publications of the same study used slow oscillating transcranial, direct-current stimulation (so-tDCS, maximum current density of 0.5 mA/cm^2^; frequency = 0.75 Hz)—that has been shown to interact with physiological slow oscillatory activity, which is typically abnormal in ADHD—over lateral prefrontal cortex during deep sleep in 12/14 ADHD adolescents compared to sham stimulation in a double-blind within patients design with 1 week break in between conditions. The outcome measures were slow oscillatory power during deep sleep in the non-stimulation periods and cognitive function improvements relative to sham tDCS in the next morning. Slow oscillatory power during deep sleep was increased, indicating an enhancement of endogenous oscillatory activity with the intervention. Cognitive improvements were in declarative memory (Prehn-Kristensen et al., 2014) and faster reaction times and decreased intra-subject variability in a motor response inhibition Go/no-go task, while no effects were observed on inhibitory processes or on intrinsic alertness and motor memory (Munz et al., 2015). The findings suggest that improvement of slow oscillatory power during sleep in lateral prefrontal regions may improve declarative memory and some EFs in ADHD.

In conclusion, the findings of the use of tDCS to improve ADHD symptoms and cognition have been mixed, with some promising results (see Table 1). Study designs and applied stimulation parameters were highly heterogeneous, hampering comparability of results. Larger and more homogeneously designed studies using a larger number of sessions of localized TDCs with and without cognitive training are needed to assess clinical and cognitive benefits. Far more knowledge is needed on the optimal stimulation parameters that can elicit clinical or cognitive efficacy, such as the optimal stimulation sites to improve ADHD symptoms or specific impaired functions, optimal stimulation amplitude, frequency of stimulation, combination of stimulation with or without cognitive training, number of sessions, etc. Children for example, have thinner skulls and less corticospinal fluid which means potentiation of the effects of brain stimulation compared to adults, and optimal dosages cannot be easily transferred from adult studies. Clear knowledge and guidance on dosage will hence be necessary for pediatric studies. Furthermore, nothing is known on the longer-term efficacy of tDCS protocols in ADHD. In healthy volunteers, up to 1 year longer-term cognitive effects have been observed of tDCS-augmented cognitive training (Katz et al., 2017). Given that tDCS is thought to affect neuroplasticity (Nitsche et al., 2008; Kim et al., 2014), potential longer-term efficacy could be the real advantage of tDCS over stimulant medication where effects are discontinued with discontinued drug administration and where effects appear to even wane in long-term continuous administration (Molina et al., 2009), presumably due to brain adaptation to the drug (Fusar-Poli et al., 2012). While direct side effects appear to be minor and transitory, such as itching and tingling over the stimulation site (Krishnan et al., 2015), there are, however, important neuroethical concerns about potential unknown negative effects of localized brain stimulation on the still developing brain. It has been suggested that the neural state at the time of stimulation (Silvanto et al., 2008) or baseline cortical excitation-inhibition levels may influence stimulation effects (Krause et al., 2013), with those with suboptimal basal neural states likely to benefit more than those who already have an optimal activation pattern. This would suggest that application in psychiatric patient groups like ADHD who have suboptimal activation patterns may be more justified than its application as cognitive enhancer in healthy children and adults. It is also possible that the stimulation of a particular region affects negatively the activation in other regions, which could then impair non-targeted functions. Inter-individual differences in traits, which may be associated with differences in baseline neural states, have in fact shown to affect the benefits or costs of brain stimulation. For example, subjects with high mathematical anxiety benefited in their reaction time to mathematical tasks with tDCS over DLPFC, while those with low mathematical anxiety had an impairment in reaction times. Also, both groups became worse in an interference inhibition task (Sarkar et al., 2014), which could possibly reflect a negative effect of tDCS of DLPFC on the neighboring IFC region which mediates interference inhibition. Inter-individual differences in brain activation at baseline are hence likely to determine whether patients benefit or not from tDCS over a particular brain region, suggesting that future brain stimulation treatment should be individualized based on baseline brain and cognitive dysfunctions. Findings of cognitive costs of tDCS on functions mediated by other brain regions are worrying. For example, prefrontal stimulation improved automaticity of learning but impaired numerical learning mediated by parietal regions while parietal stimulation impaired automaticity of learning mediated by prefrontal regions and improved numerical learning (Iuculano and Cohen Kadosh, 2013). It will therefore be crucial to assess potential costs on non-targeted cognitive functions which may occur via indirect downstimulation of other brain regions that are interconnected with the stimulated site and that mediate these non-targeted functions. This knowledge will be crucial to understand the cost-benefits of localized brain stimulation to the individual patient, and in particular to children who have higher brain plasticity. Most ethical considerations have concluded that there are no ethical reasons against tDCS in children and adolescents who have a medical condition that is handicapping and where potential side effects can be outweighed by benefits, while use of tDCS as cognitive enhancer in healthy children and adolescents is not advised (Cohen Kadosh et al., 2012; Palm et al., 2016). These benefits and costs, however, will still have to be established in ADHD as well as in other childhood disorders.

## Neurofeedback Using Real-Time fMRI and NIRS

**Figure 3:**
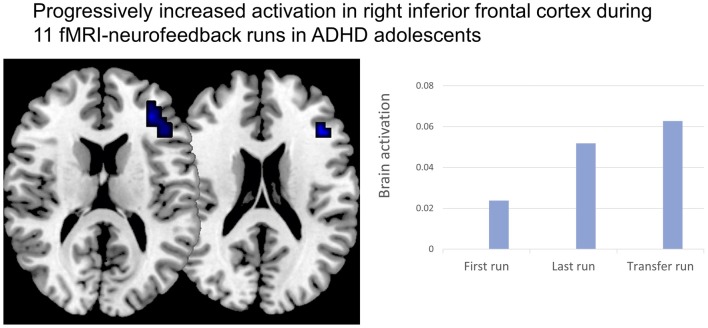
Increased activation in right IFC in 18 ADHD adolescents after 11 runs of fMRI Neurofeedback (NF) compared to controls who had to self-regulate another region. The patients also showed transfer effects (self-regulation without NF) in the same region (Alegria et al., 2017).

NF is an operant conditioning procedure that, by trial and error, teaches participants to volitionally self-regulate specific regions or networks through real-time audio or visual feedback of their brain activation which can be represented on a PC. For children this can be gamified in an attractive way. Given that ADHD is typified by poor self-control (Schachar et al., 1993), and enhancing brain-self-control is the target of NF, ADHD is the psychiatric disorder where NF has been most applied, using electrophysiological neurofeedback (EEG-NF), targeting abnormal EEG biomarkers such as theta/beta rhythms or slow cortical potentials. Despite the fact that EEG-NF has been tested in ADHD for over 50 years, the latest meta-analyses of randomized controlled trials of EEG-NF show medium effect sizes for symptom improvements (Arns et al., 2009), reduced to trends when only “probably” blinded raters are included (Murray et al., 2013; Cortese et al., 2015). Interestingly, however, unlike psychostimulant treatment, NF effects seem stable and longer-lasting (up to 2 years), with no side effects (Gevensleben et al., 2010; Arns et al., 2014). In ADHD studies, 30–40 h runs are commonly used in EEG-NF (Arns et al., 2009). Real-time functional magnetic resonance imaging-NF (rtfMRI-NF) teaches subjects to self-regulate blood-oxygen level-dependent (BOLD) response in specific brain regions based on real-time feedback of this response. The BOLD self-regulation can be achieved in less than 40 min (Thibault et al., 2016) in healthy adults. While more sessions may be needed to achieve clinical efficacy in patient groups like ADHD, the ability to learn to self-regulate brain activation appears to be much faster with rtfMRI-NF than EEG-NF (Thibault et al., 2016), possibly due to superior signal to noise ratio and superior spatial localization specificity of the fMRI signal. Furthermore, fMRI-NF can modulate activation in deep cortical or subcortical underfunctioning regions in ADHD found in fMRI meta-analyses, such as right IFC or the basal ganglia (Norman et al., 2016). In other psychiatric and neurological disorders rtfMRI-NF has shown some clinical potential (Thibault et al., 2015, 2016), showing generalization to NF-free transfer runs and longer-term beneficial effects on cognitive and behavior symptoms of up to several months (Zilverstand et al., 2015). Importantly, by learning to self-upregulate isolated regions, participants learn to co-regulate other areas interconnected with the target region, suggesting modulation of entire networks (Emmert et al., 2016; Thibault et al., 2016). Despite the potential of rtfMRI-NF in ADHD patients, only two studies have been conducted in the disorder. The first feasibility pilot study tested rtfMRI-NF of dorsal anterior cingulate cortex (dACC) over 4 h fMRI-NF sessions in combination with performance on a mental calculation task expected to increase dACC activation in seven adults with ADHD compared to six adults with ADHD who performed the same training task in the MRI scanner but did not receive rtfMRI-NF (Zilverstand et al., 2017). The study found that although both groups showed similar dACC activation increases during training and transfer runs, ADHD symptoms were not improved in either group. Only the active, but not the control group showed performance improvements in sustained attention and WM, suggesting some superior effects of rtfMRI-NF on cognitive performance. The study was underpowered to test potential clinical benefits. However, it showed first evidence of feasibility of rtfMRI-NF in ADHD adults (Zilverstand et al., 2017). The second study from our lab tested rtfMRI-NF in 31 ADHD adolescents between 12 and 17 years (Alegria et al., 2017). The study had two active treatment conditions: the target group (*N* = 18) had to learn to upregulate the rIFC, while the control group (*N* = 13) had to upregulate a control region, the left parahippocampal gyrus (lPHG). Both groups were tested before and after treatment in clinical and cognitive measures as well as in an fMRI Stop task and were followed up a mean of 11 months later in the main ADHD clinical outcome measure. Participants were trained to enhance activation of the target/control regions in 11 sessions of 8.5 min of rtfMRI-NF over four scan hours over 2 weeks. They were trained on a computer game where a rocket moved towards the sky, passing through clouds, and ultimately reaching some planets every time they managed to increase the activation of the target/control region. The fMRI data showed significantly enhanced activation as well as enhanced linear activation increase in two regions of the rIFC across all 11 sessions in the active relative to the control group and enhanced linear activation increase in three regions of the lPHG in the control relative to the active group (Figure 3). Only the active group, however, showed significant transfer effects (increased activation in the target region when no feedback was provided), which furthermore was significantly associated with the reduction of clinical ADHD symptoms (Figure 3). Both groups improved significantly in the main outcome measures, the parent-rated clinical ADHD severity measures, with no group by treatment interaction effects. Behavioral changes were furthermore significantly correlated with the linear activation increases in IFC in the active group, thus demonstrating brain-behavior associations. Effect sizes were medium size at post-assessment, but large in the active group (almost 1) at 11 months follow-up with an 26% reduction in ADHD symptoms, while only trend-level significant medium effect size changes of 16% were observed in the control group at follow-up, suggesting longer-term and potentially delayed consolidation effects of fMRI-NF in the active group (Alegria et al., 2017). In addition, only the active group showed cognitive performance improvements such as trend-level reduced commission errors on a sustained attention task. Furthermore, the active compared to the control group showed significantly enhanced activation in rIFC in the Stop task after the fMRI-NF training compared to before, comparable to the rIFC upregulation effects we have previously observed with stimulant medication during the same task (Rubia et al., 2011b, 2014b; Cubillo et al., 2014). While the active treatment had significant advantages over the control treatment, such as stronger brain-behavior correlations, exclusive transfer and cognitive effects and exclusive brain activation benefits in the stop task, the lack of a sham control condition makes it impossible to rule out potential placebo effects. Alternatively, is also possible that the control condition was “too active” and hence also elicited positive behavioral changes. Whole brain analyses reinforce this view by showing that the active group, in addition to right IFC, activated bilateral DLPFC and striato-insular cognitive control networks, while the control group activated posterior temporal and parahippocampal/occipital regions. It is thus possible that the active group benefitted from trained rIFC-striato-insular activation increase, while the control group benefitted from trained activation increase in posterior visual-spatial attention regions connected to lPHG, both of which are relevant to ADHD and could have accounted for the behavioral benefits (Rubia et al., 2014a; Norman et al., 2016; Alegria et al., 2017). The proof-of-concept study in adolescents with ADHD thus suggests that rtfMRI-NF of rIFC is feasible, safe, transferrable and has short-term and even stronger longer-term efficacy in reducing ADHD symptoms. Furthermore, it is associated with better inhibitory rIFC activation and trend-level improvements in attention performance. However, replication in larger samples and a comparison with a non-active sham-NF placebo control condition will be needed to establish clinical efficacy and to rule out placebo effects.

A pilot study tested the related neural hemeodynamic modulation method of NIRS Neurofeedback (NIRS-NF) of the left DLPFC in nine ADHD children, compared to EEG-NF (*N* = 9) and electromyography-NF (*N* = 9). Only NIRS-NF resulted in significant improvements in clinical ADHD symptoms and in cognitive inhibition and attention functions after 11 h sessions over 4 weeks, which was, however, not superior to EEG-NF or electromyography-NF (Marx et al., 2015).

In conclusion, some of the findings of these small proof of concept studies using fMRI-NF and NIRS-NF are promising. However, larger, double-blind, placebo-controlled randomized controlled trials need to further assess the potential efficacy of fMRI or NIRS-NF in ADHD. Similar to the issues raised above for the brain stimulation field, in rtfMRI-NF or NIRS-NF nothing is known on optimal number of NF sessions, whether there is a saturation or a plateau of self-regulation in specific brain regions, after how many sessions, or how and which interindividual differences affect learning of brain self-regulation. Also, transfer effects on clinical behavior are unclear. Other untested questions are optimal reinforcement strategies or cognitive strategies when applying fMRI or NIRS-NF in children. Also, while in the field of brain stimulation concerns have been raised on potential costs of brain stimulation of a specific region on non-stimulated regions, positive or negative side effects on non-stimulated regions or non-targeted cognitive functions has never been addressed in NF studies. It is entirely possible that the self-regulation training of a particular brain region has a downregulation effect on neighboring, interconnected or contralateral regions and the potential costs of such downregulations need to be assessed. In fact, our rtfMRI-NF study in adolescents with ADHD, for example, showed a reduction in the active rIFC group in activation of the parahippocampal control region, while the control group had a decrease in right IFC activation, suggesting that the self-regulation of a particular region leads to the downregulation of other regions (Alegria et al., 2017).

One of the key positive findings from all NF modalities, including EEG-NF, NIRS-NF and fMRI-NF, is evidence for longer-term delayed consolidation effects which appear to be more pronounced at follow-up than at post-NF assessments (Arns and Strehl, 2013; Arns et al., 2014; Marx et al., 2015; Alegria et al., 2017). Such delayed consolidation effects reinforce the notion that brain self-regulation via NF affects neuroplasticity and may hence have unique longer-term efficacy compared to stimulant medication which does not affect neuroplasticity and may even loose efficacy over time (Molina et al., 2009), due to potential brain adaptation (Fusar-Poli et al., 2012). In fact, neuroplasticity of NF has been demonstrated in humans in the form of cortical excitability changes, white matter tract and structural changes (Sitaram et al., 2017). The stability of these changes over time, is, however, unknown. This potential for longer-lasting neuroplastic effects and the apparent lack of side effects are likely to be the main attraction of NF therapies.

## Overall Conclusions

In conclusion, there is relatively consistent evidence from several meta-analyses of fMRI studies of “cool” EF, that ADHD patients have cognitive-domain dissociated deficits in several neural networks that mediate higher-level cognitive functions, including different right and left hemispheric fronto-striato-thalamic and fronto-parieto-cerebellar networks such as IFC-ACC-SMA-striato-thalamic networks for inhibitory control, right DLPFC-parieto-striato-cerebellar networks for attention functions, bilateral DLPFC and ACC regions for WM and left IFC-parieto-cerebellar networks for timing functions (Cortese et al., 2012; Hart et al., 2012, 2013; McCarthy et al., 2014; Lei et al., 2015; Norman et al., 2016; see Figures 1, 2). The findings point towards complex multisystem impairments in several dorsal and ventral fronto-striato-parietal and fronto-cerebellar networks that mediate these functions. The fMRI literature, however, is characterized by a bias favoring cool EF fMRI paradigms. There is emerging evidence for abnormalities in ADHD in regions that mediate motivation control during hot EF and emotion processing, most prominently the ventral striatum, but also limbic and orbitofrontal/vmPFC-limbic areas (see Figure 2). In addition, there is consistent evidence for ADHD patients to have problems to deactivate the DMN, suggesting an abnormal interrelationship between hypo-engaged task-positive cognitive networks and a poorly “switched off” DMN, both of which are likely responsible for impaired cognitive performance (Cortese et al., 2012; Hart et al., 2012, 2013; Christakou et al., 2013; Figure 2).

The majority of fMRI studies have focused on the male, combined hyperactive-impulsive/inattentive combined ADHD subtype. Future studies will need to focus on understanding the (differential) neurobiological basis of different ADHD subtypes such as inattention without hyperactivity, or ADHD with emotional dysregulation. Furthermore, more understanding is needed on comorbid cases with other disorders such as autism, anxiety and affective disorders as well as on females with ADHD and gender differences. Future studies therefore ideally should be longitudinal, multimodal and tied to epidemiological samples.

Clinical translation of neuroimaging is still in its infancy in the field of ADHD. Pattern recognition analyses applied to functional (or structural) imaging data to make individual predictions on diagnostic status are promising, but more so for homogenous subtypes that likely share the same “biotype” rather than heterogenous large groups of ADHD patients with different comorbidities or medication status. They will need to show replicability and clinical utility which will be the challenge over the next decades.

Several brain stimulation studies with heterogeneous study designs have been conducted in small groups of ADHD children and adults, most of them using tDCS in either single or five sessions targeting mostly DLPFC or IFC based on the fMRI studies conducted in ADHD over the last two decades. The findings show some improvements on clinical symptoms or selective cognitive functions, with, however, also negative findings. Larger sham-controlled studies are needed to further test the efficacy of tDCS and potential costs on non-targeted cognitive or behavioral functions. In addition, far more knowledge is needed on the optimal stimulation protocols for different age and patient subpopulations (i.e., stimulation site, strength, frequency, number of sessions, etc). It is likely that brain stimulation combined with cognitive training has a larger potential to enhance brain plasticity in ADHD than brain stimulation alone. This will also require the development of good cognitive training tasks that target ADHD-relevant functions to be used in combination with brain stimulation techniques. Given minimal side effects, tDCS is a promising tool for the treatment of childhood onset psychiatric disorders, since it provides the opportunity to positively influence atypical brain development early and persistently (Krause and Cohen Kadosh, 2013). However, there is some worrying evidence for potential costs of localized brain stimulation on other, non-targeted functions and these need to be thoroughly investigated before clinical application.

NF studies using higher spatially resolved neuroimaging techniques such as NIRS and rtfMRI have only recently been piloted in ADHD, showing feasibility but mixed findings in relatively small subject numbers. Larger, sham-controlled studies that allow the identification of predictors of learning are necessary to establish whether NIRS or fMRI NF training has potential as a treatment for some individuals with ADHD.

In conclusion, the field of cognitive neuroscience in ADHD, like in other disorders, has opened up to translational neuroscience studies in an attempt to use functional neuroimaging data for diagnostic classification purposes or as biomarkers for treatment. Neurotherapeutics seem attractive for ADHD due to their safety and minimal or no side effects compared to medication treatments, and due to their potential for longer-term neuroplastic effects, which drugs cannot offer. However, neurotherapies need to be more thoroughly tested for their short- and longer-term efficacy, optimal “dose” effects (i.e., optimal frequency/strength of stimulation or number of stimulation/NF sessions), potential costs that may accompany the benefits, and their potential for individualized treatment (which ADHD subtype responds to which neurotherapy and why). It is likely that different subgroups of ADHD patients will benefit from either NF, brain stimulation or medication and establishing this knowledge will be crucial to the benefit of individual patients.

## Author Contributions

The author confirms being the sole contributor of this work and approved it for publication.

## Conflict of Interest Statement

The author has received grants from Lilly and Shire and speaker’s honoraria from Shire, Lilly and Medice.

## Footnotes

^1^https://login.webofknowledge.com
